# Electrical Switchability and Dry-Wash Durability of Conductive Textiles

**DOI:** 10.1038/srep11255

**Published:** 2015-06-12

**Authors:** Bangting Wu, Bowu Zhang, Jingxia Wu, Ziqiang Wang, Hongjuan Ma, Ming Yu, Linfan Li, Jingye Li

**Affiliations:** 1 CAS Center for Excellence on TMSR Energy System, Shanghai Institute of Applied Physics, Chinese Academy of Sciences, Shanghai 201800, China; 2 University of Chinese Academy of Sciences, Beijing 100049, China

## Abstract

There is growing interest in the area of conductive textiles in the scientific and industrial community. Herein, we successfully prepared a conductive textile via covalently grafting polyaniline (PANI) onto cotton by a multi-step treatment process. The conductivity of the resultant fabric could be tuned by immersing in water having different pH values. The conductive and insulating properties of the textile could be conveniently switched by alternately immersing in acidic and alkaline bath solutions. Most importantly, the resultant conductive fabrics were able to withstand 40 simulated dry-wash cycles, with almost no decay in the electrical conductivity, indicating their excellent dry-wash durability. The present strategy for fabricating conductive fabrics with excellent switchability of electrical properties and dry-wash durability is expected to provide inspiration for the production of multifunctional conductive textiles for use in hash or sensitive conditions.

Conductive textiles are currently used in the fabrication of pressure sensors^1^, antenna^2^, electromagnetic interference (EMI) shielding devices^3^, flexible heaters, static control clothing^4^,^5^, and so on. In addition, conductive textiles have been deemed as being crucial for applications in next-generation wearable consumer electronics and smart clothing, which integrate the intelligent functionality of electronic devices with the flexibility and comfort of stylish clothing and are being designed to meet various innovative applications in military, public safety, healthcare, space exploration, sports, and consumer fitness fields^6^,^7^,^8^. Generally, conductive textiles can be made by weaving metal strands into the construction of the textiles^9^, or by coating (depositing) or embedding electrically conductive components^10^, often carbon^11^, nickel, copper, gold, silver, or titanium on the surface of textiles. Although these composite textiles have excellent conductivities, the stiff-feeling and easy-decay, which may be due to the breakage of wires, sloughing of depositions, or chemical corrosion during use, are drawbacks that limit their applications.

Conductive polymers have gained much attention since their discovery, as they possess excellent electrical, electronic, magnetic, and optical properties commonly associated with metals, in addition to retaining the light-weight, flexibility, and processability of conventional polymers^12^,^13^. Among the various conductive polymers, polyaniline (PANI) has been of great interest to many researchers owing to its lightness, good environmental and thermal stability, high electrical conductivity, good optical properties^14^, easy preparation, and economic affordability^15^,^16^. Furthermore, its conductivity can be modulated by carefully controlling its oxidation state and protonation level^17^,^18^,^19^, which makes PANI a prime choice in the fabrication of multifunctional conductive materials. In view of their soft nature, coating conductive polymers such as PANI onto fabric substrates is a feasible and effective strategy to overcome the problem of stiff-feel and chemical corrosion associated with conventional metal/fabric blends^20^,^21^. However, the problem of conductivity decay, which is caused by the weak physical attachment between the conductive coating and fabric substrates, remains unsolved^21^. In practice, fabrics get inevitably stained by substances such as dust and dirt. Mechanical scrubbing and soaking in chemicals during the washing and cleaning processes could damage the coatings on the conductive layers or break metal wires, resulting in conductivity decay^22^. Therefore, it is necessary to improve the washing durability of conductive textiles by enhancing the connection of the conductive ingredients with the fabric substrates, which would improve the longevity of conductive textiles.

It is known that the modification of polymer substrates by grafting is a strong approach in the fabrication of stable functional materials owing to the covalent bonding achieved between the functional moieties and the substrates^23^,^24^,^25^,^26^. In our previous work, we successfully fabricated novel superhydrophobic fabrics^27^,^28^,^29^, antibacterial fabrics^30^,^31^, and self-cleaning fabrics^32^ with excellent washing durability and abrasion resistance via gamma ray irradiation-induced graft polymerization of functional monomers and components. H. Lin *et al*. also prepared a washing-durable antibacterial fabric by grafting amino-terminated hyperbranched polymer functionalized with silver nanoparticles^33^. Herein, we report a washing-durable conductive textile prepared through the graft polymerization of aniline onto a cotton fabric via multi-step treatment. An overall schematic diagram of the process is depicted in Fig. 1 and details of each treatment step are provided in the ***Experimental Section*** in the Supplementary information. For brevity, we designate PGC fabric, APGC fabric and APGC-g-PANI fabric as the cotton-g-PGMA fabric (*i.e*. cotton grafted with polyglycidyl methacrylate), 4-aminophenethylamine (APA) functionalized PGC fabric and APGC fabric grafted with PANI, respectively. Briefly, PANI is covalently linked to cotton fabric substrates though in-situ growth from the phenylamino end of APA on APGC fabric, forming a graft layer to cover the surface of the fibers in the fabric. Special mention should be made of this route avoids the various drawbacks of solid PANI, including insolubility in most solvents and infusibility and poor machinability^34^, which hinder its applications.

## Results and Discussion

**Figure 2:**
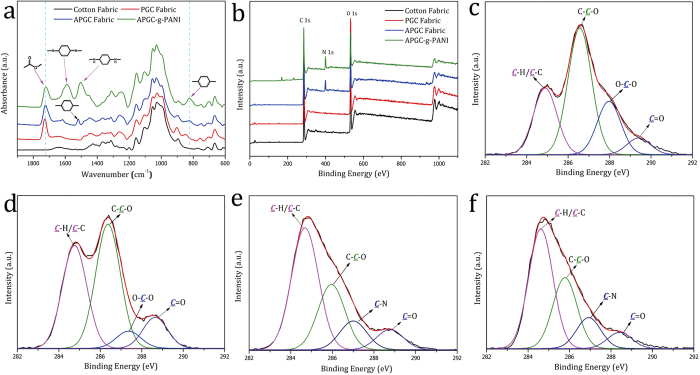
(**a**) FTIR-ATR spectra and (**b**) full-scan XPS spectra of cotton fabric, PGC fabric, APGC fabric and APGC-g-PANI fabric; C1s XPS spectra of (**c**) cotton fabric, (**d**) PGC fabric, (**e**) APGC fabric and (**f**) APGC-g-PANI fabric. The DG of PGMA in PGC fabric is 25.6 wt. %, the DG of PANI in APGC-g-PANI fabric is 7.3 wt. %. About 24.7% of expoxy group is reacted with APA in APGC fabric.

Figure 2a shows the Fourier transform infrared with horizontal multi-rebound attenuated total reflection (FTIR-ATR) spectra of the fabrics obtained from each treatment step. The presence of the absorption peak at 1722 cm^−1^, which is assigned to the C = O stretching vibration, demonstrates that polyglycidyl methacrylate (PGMA) chains have been successfully grafted onto the cotton fabric via gamma-ray irradiation-induced polymerization. The peaks at 1518 cm^−1^ and 823 cm^−1^, which are assigned to the stretching vibration and deformation vibration of the para-substituted benzene ring on the APGC fabric, respectively^35^,^36^, indicate that APA is attached to the PGMA chains during the amino-epoxy ring cleavage reaction. Evidence of the grafting of PANI on the APGC fabric (APGC-g-PANI fabric) is obtained by the appearance of obvious peaks at 1583 cm^−1^ and 1492 cm^−1^, which are attributed to the aromatic ring breathing of the nitrogen quinoid structure and benzoid ring in aniline oligomers, respectively^35^,^37^. This was also confirmed by the results of the X-ray photoelectron spectroscopy (XPS), where the surface chemical composition of various samples were analyzed. Figure 2b and Table S1 show that the carbon content in the sample increases, whereas the oxygen content decreases, over the course of the grafting treatments. Moreover, the presence of nitrogen is detected after the APA attachment step. By comparing the C1s spectra of the cotton fabric obtained after each treatment step with that obtained before and after treatment, the intensity of the C-C/C-H peak is found to increase after each step, whereas the intensities of the C-O and O-C-O peaks are attenuated (Fig. 2c–f), since the CH_2_ and cyclobenzene contents are increased with PGMA and PANI grafting. The appearance of the C-N peak around 287 eV in Fig. 2e,f confirms the presence of APA and PANI on the cotton substrates. From the N1s spectra of APGC and APGC-g-PANI fabric shown in Figure S1a&b, the intensity of the C-***N***-H peak at 401.2 ± 0.2 eV is found to decrease after the grafting of PANI on the fabric. The decrease in the intensity of the N-H peak also indicates that aniline was partly oxidized into the quinoid structure during the in-situ redox polymerization on the fabric, which would impart electrical conductivity to the resultant fabric after doping.

**Figure 3:**
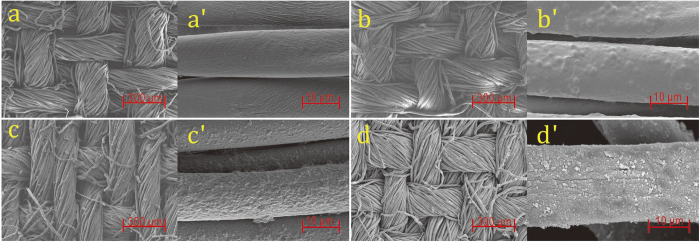
SEM images of (**a**,**a‘**) pristine cotton fabric, (**b**,**b‘**) PGC fabric, (**c**,**c‘**) APGC fabric and (**d**,**d‘**) APGC-g-PANI fabric.

From the scanning electron microscopy (SEM) images shown in Fig. 3, it is evident that the micromorphology of the cotton fabrics changed after each treatment step. The natural wrinkles and creases on the pristine cotton fiber surfaces (Fig. 3a,a’) were found to be covered and smoothed with a layer of grafted PGMA after the gamma ray irradiation-induced graft polymerization (Fig. 3b,b’). In addition, a rough surface was formed by APA anchoring (Fig. 3c,c’), which may be explained by the cleavage of the epoxy groups and the aminoethyl group from the APA addition on the grafted PGMA chains. Finally, the surface of the fibers changed to a granular microstructure (Fig. 3d,d’) as a result of the supramolecular assembly of the PANI macromolecules growing from the phenylamino end of the APGC fabric^36^. Further, the fibers of the fabric were found to be finely covered by the grafted PANI layer, which would impart electrical conductivity to the cotton fabric.

**Figure 4:**
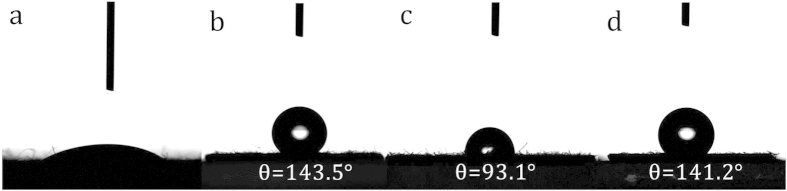
Photographs of water contact angle of (**a**) pristine cotton fabric, (**b**) PGC fabric, (**c**) APGC fabric and (**d**) APGC-g-PANI fabric.

We also investigated the changes in the surface properties of the fabrics by contact angle analysis at each step and the results are shown in Fig. 4. Cotton is a well-known hydrophilic natural polymeric material. When a droplet of water was placed on the cotton surface, it was quickly soaked into the fabric (Fig. 4a). After the grafting of PGMA, the water contact angle of the PGC fabric was 143.5° (Fig. 4b), which indicates good hydrophobicity. On the APGC fabric, the water contact angle reduced to 93.1° owing to the epoxy cleavage and amino addition reaction (Fig. 4c). However, after the in-situ redox graft polymerization of aniline that resulted in APGC-g-PANI fabric, the water contact angle of the surface increased again to 141.2° (Fig. 4d). The results demonstrate that good hydrophobic properties were imparted to the cotton fabric by treating through this route. It is well-known that the water or humidity in the air exerts a significant influence on the charge decay and conductivity stability of conductive materials, including antistatic coatings, static dissipative materials, and electrets^38^,^39^. Therefore, the good hydrophobicity of the fabrics would enhance their humidity resistance properties and suppress conductivity decay^40^.

**Figure 5:**
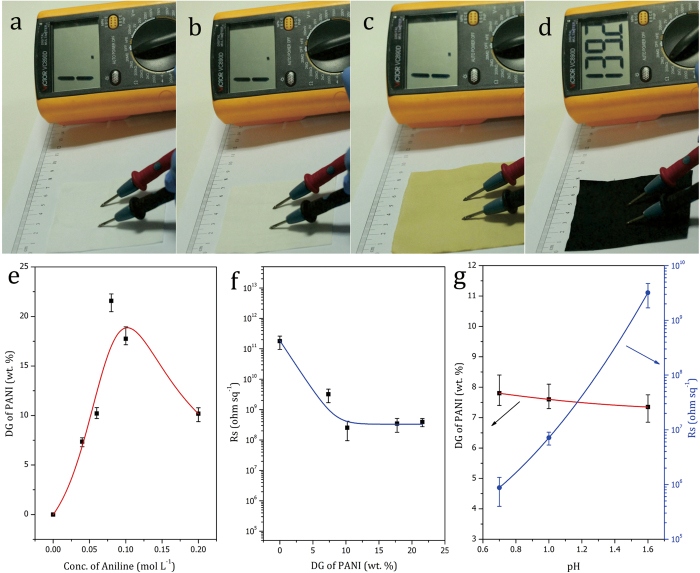
Digital images and preliminary conductivity tests of (**a**) cotton fabric, (**b**) PGC fabric, (**c**) APGC fabric, and (**d**) APGC-g-PANI fabric with a DG of 7.3 wt.%; (**e**) Effect of aniline concentration on the values of DG of PANI; (**f**) Effect of DG of PANI on the conductivity of APGC-g-PANI fabric; (**g**) Effect of the pH of the reactant solution on DG of PANI and conductivity of APGC-g-PANI fabric.

From Fig. 5a–d, it is evident that the color of the samples changed successively from white to light gray to yellow to dark green. Preliminary tests with a digital multimeter showed that the pristine cotton fabric, PGC fabric, and APGC fabric were electroinsulating, whereas the APGC-g-PANI fabric exhibited apparent conductivity. This result illustrates that PANI was grafted on the fabric uniformly and a continuous conductive network was formed between fibers throughout the fabric by the PANI graft chains. Although redox polymerization of aniline is well-established, the grafting of PANI on the cotton fabric is a heterogeneous reaction, and therefore, the diffusion of aniline molecules in solution towards the fabric substrate surface is critical for obtaining a desired degree of grafting (DG) of PANI. Therefore, the aniline concentration was controlled by feeding drop-wise and the reaction was allowed to proceed under intense mechanical agitation in an ice-water bath. An equal concentration of oxidant, *i.e*. ammonium persulfate (APS), was added into the reactor in the same manner. The details are provided in the ***Experimental Section S4*** in the Supplementary Information. Figure 5e shows that the DG of PANI in the APGC-g-PANI fabric increases rapidly with an increase in the concentration of aniline up to 0.1 mol L^−1^. The DG of PANI is around 20 wt.%, when the aniline concentration at 0.1 mol L^−1^, which is much higher than that (~9 wt.% ) reported previously^41^. It indicates this multistep route is very effective to graft PANI on common substrates. A further increase in the aniline concentration results in a decrease in the DG, due to the simultaneous occurrence of homo-polymerization and graft polymerization in the system. The high concentration of aniline would cause most of the aniline to be homo-polymerized before diffusing onto the cotton fiber, which would hinder the growth of the graft chain. The electrical conductivity measurements of APGC-g-PANI fabrics with different DG values show that the surface resistance of the fabrics decrease from ~5 × 10^11^ ohm sq^−1^ to ~2 × 10^9^ ohm sq^−1^, at DG values as low as 7.3 wt. % (Fig. 5f), which shows the electrical conductivity increase of the resultant APGC-g-PANI fabrics. It demonstrates a continuous conductive circle has been formed across the entire fabric because the cotton fibers of fabric have been completely covered by PANI grafting layer (Fig. 3d,d’). When the DG of PANI increased to 10.2 wt. %, the surface resistance decreased to ~3 × 10^8^ ohm sq^−1^. Interestingly, there is no obvious increase in the conductivity of the fabrics when the DG further increases after 10.2 wt. %, suggesting that the thickness of grafting PANI layer on surface of the fibers has been able to overcome the size effect of conductive layer when DG of PANI exceeds 10.2 wt. %.

It is known that the conductivity of PANI is mainly related to the redox state and doping level^17^,^18^,^19^. In this study, we investigated the effect of pH of the reactant solution on the DG of PANI and the conductivity of the APGC-g-PANI fabrics. The results are shown in Fig. 5g. It may be observed from the figure that the DG of PANI in the APGC-g-PANI fabric remains constant at around 7.5 wt. % as a function of pH, indicating that the change in the pH of the reactant solution has almost no effect on the graft polymerization of aniline. In contrast, the conductivities of the APGC-g-PANI fabrics increase rapidly with a decrease in the pH of the reactant solutions, as a result of the increase in the degree of protonation (H^+^ doping) of the grafted PANI with an increase in the H^+^ concentration (*i.e*., decrease in pH), which results in a decrease in the surface resistance.

**Figure 6:**
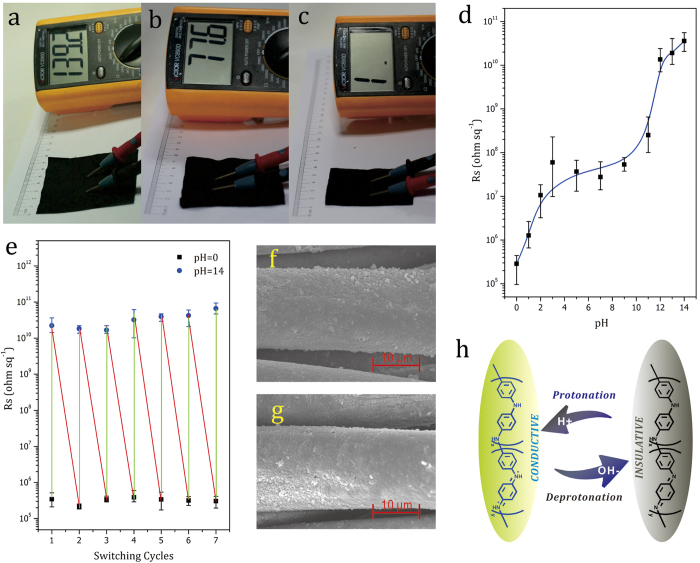
Digital images and preliminary conductivity measurements of (**a**) pristine APGC-g-PANI fabric (DG = 7.3 wt.%), (**b**) APGC-g-PANI fabrics immersed in acidic (pH = 0) and (**c**) alkaline (pH = 14) solutions; (**d**) conductivity of the APGC-g-PANI fabric after immersing in bath solutions with different pH values (DG = 10.2 wt.%); (**e**) Cyclic conductivity switching test of the APGC-g-PANI fabric conducted at pH values of 0 and 14; (**f**) SEM image of APGC-g-PANI fabric (DG = 7.3 wt.%) after immersion in acidic (pH = 0) solution; (**g**) SEM image of APGC-g-PANI fabric after immersion in alkaline (pH = 14) solution; (**h**) schematic showing the transformation between emeraldine base (insulating form) and emeraldine salt (conductive form) by H+ protonation and OH− deprotonation of PANI chains.

Motivated by the results of the study of the effect of the reactant solution pH on the conductivity of APGC-g-PANI fabrics (Fig. 5g), we conceived a post-preparation strategy to tune the electrical conductivity of the APGC-g-PANI fabrics. From Fig. 6a–c, it can be seen that the conductivity of the APGC-g-PANI fabric improved after it was immersed in an HCl bath solution with a pH of 0, whereas the fabric turned insulating after it was immersed in a NaOH bath solution with a pH of 14. We further investigated the effect of pH of the bath solution on the conductivity of the APGC-g-PANI fabrics and the results are shown in Fig. 6d. The surface resistance increases rapidly as the pH is increased from 0 to 3. Between pH values of 3 and 9, the surface resistance remains constant, beyond which another increase in the surface resistance is seen from pH 9 to pH 14, illustrating a good correlation between the pH of the bath solution and conductivity. Furthermore, when the APGC-g-PANI fabric was alternately immersed in HCl (pH = 0) and NaOH (pH = 14) bath solutions, the fabric shows a reversible switch between conductive and insulating behavior (Fig. 6e). As seen from the SEM images in Fig. 6f,g, no damage is seen on the PANI layer after immersion in the acidic and alkaline solutions, when compared with the image of pristine APGC-g-PANI fabric (Fig. 3d’). These results can be explained by the reversible equilibrium between H^+^ protonation and OH^−^ deprotonation of PANI under different external pH conditions, which cause the conductivity switching behavior. As reported previously, the PANI structure can reversibly switch between the emeraldine base (insulating form) and emeraldine salt (conductive form) by acidic doping and alkaline de-doping treatments^42^,^43^,^44^,^45^, as shown in Fig. 6h. This conclusion is also confirmed by comparison with FTIR-ATR spectra of APGC-g-PANI fabric before and after immersing in acidic and alkaline bath solution in Figure S2. It can be observed that the characteristic peaks of aromatic ring breathing of the quinonoid and benzenoid rings shift to 1589 and 1512 cm^−1^ after immersing in alkaline solution, which suggests PANI was de-doped with alkaline immersing^37^. And these two peaks shift back to 1583 and 1489 cm^−1^ after doping with acidic bath solution. Based on this, we can easily tune and switch the fabric conductivity by immersion in acidic or alkaline bath solutions. Furthermore, the sensitivity of these fabrics to pH could be used in applications such as flexible sensors for testing the presence of ammonia, acetic acid, HCl, H_2_S, and so on^43^,^44^.

**Figure 7:**
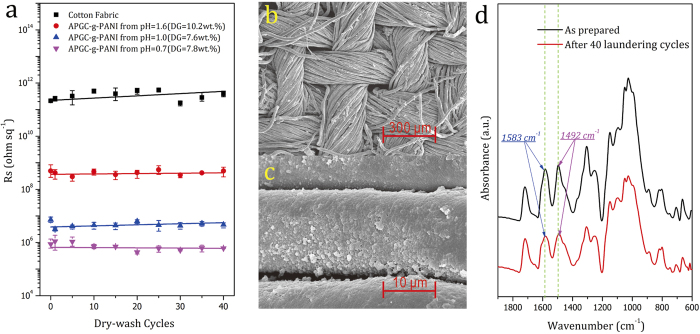
(**a**) Conductivity tests of pristine cotton fabric and APGC-g-PANI prepared from systems with different pH values after simulated dry-wash; (**b**,**c**) SEM images of APGC-g-PANI after 40 dry-wash cycles; (**d**) FTIR-ATR spectra of APGC-g-PANI fabric before and after 40 times of simulated dry-wash in tetrachloroethylene (TTE).

As mentioned before, conductive textiles consisting of nonconductive fabric substrates and interwoven metal wires or coated conductive polymer layers suffer from poor durability during conditions of actual use, such as washing, folding and rubbing, which induce conductivity decay^22^. To achieve durable conductive textiles, we grafted the conductive polymer, PANI, onto the fibers. For evaluating the washing durability of the resultant conductive fabrics, we carried out a simulated dry-wash test using tetrachloroethylene (TTE), based on the standard test methods 132-2004 and 86-2005 recommended by AATCC (American Association of Textile Chemists and Colorists). The details of the tests can be found in the ***Methods***. Figure 7a shows the surface resistance of APGC-g-PANI fabrics prepared at different pH values during the simulated dry-wash test. The surface resistance of the samples was observed to be very stable even after 40 dry-wash cycles. Figure 7b,c show no obvious destruction of the PANI layer on the fiber after the dry-wash, indicating that the conductive network of the APGC-g-PANI fabric was able to withstand the washing process. We also investigated the chemical information by FTIR-ATR spectroscopy, which shows no obvious change of APGC-g-PANI fabric after 40 times of dry-wash by comparison with before (Fig. 7d). These evidences undoubtedly substantiate that chemical grafting conductive components, such as PANI, onto common textiles is an effective route to prepare conductive textiles with enhanced stability in practice.

In conclusion, a conductive textile has been successfully prepared by covalently grafting PANI onto a cotton fabric substrate. When the DG of PANI was just 7.3 wt. %, the surface of the fibers in the fabric was covered completely and a conductive network was formed. The conductivity of the resultant conductive fabrics was significantly influenced by the pH of the reactant solution. In addition, the conductivity of the fabric could also be tuned post-preparation by immersing in bath solutions of different pH values. A switch between conductive and insulating behavior was achieved by immersing in acidic and alkaline solutions, respectively, post-preparation. The dry-wash tests demonstrated that the conductive fabrics prepared by covalently binding PANI chains on common fabrics are able to withstand the dry-wash process and exhibit highly stable conductivity values without any decays. In view of the outstanding electrical switchability and dry-wash durability, we believe that conductive textiles prepared by this technique will undoubtedly become promising candidates for applications in the fabrication of various functional fabric-based electrical devices.

## Methods

### Conductivity Tests

The tests were carried out according to a standard method (AATCC Test Method 76-2005) which is proposed to evaluate the electrical surface resistivity of fabrics. The samples were conditioned at a relative humidity of 65 ± 2% and a temperature of 20 ± 1 °C for 1 h before the electrical conductivity measurements were performed. The conductivity tests were carried out using an ultrahigh-resistivity meter (Hiresta-UP MCP-HT450) with a URS probe electrode at a voltage of 10 V. A ring electrode probe consisting of an inner electrode and outer electrode (as shown in Figure S3) was directly placed on the samples for the measurements. At least 5 different locations on each sample were measured.

### Conductivity Tuning and Switching Tests

APGC-g-PANI fabrics were washed with an abundance of deionized water and completely dried before use. The dry fabrics were soaked in a bath solution at a certain pH at room temperature (about 25 °C) for 30 min with intense magnetic stirring. Thereafter, the fabrics were washed with DI water, placed in a ventilated hood, and oven-dried to a constant weight. The fabrics were immersed in a bath solution that was at a different pH value compared to the first cycle, and subjected again to the above procedures. The pH values of the bath solutions were adjusted using HCl and NaOH and the fabrics were immersed in solutions of increasing pH values from 0 to 14. Next, the fabrics were subjected to electrical switchability testing. In these tests, the samples were first immersed in a NaOH solution (pH = 14) at room temperature for 30 min with intense stirring, and then washed with abundant water and dried. Conductivity of the samples was measured using the same procedure described above. After the measurements, the samples were treated in HCl solution (pH = 0) and the post-immersion procedures were followed as described previously. Several such cycles were conducted by alternately treating in bath solutions with pH values of 0 and 14.

### Dry-Wash Tests

The evaluation method used was a simplified version of the standard test methods 132-2004 and 86-2005 recommended by AATCC. The samples were cut into 5 cm × 5 cm (2 in. × 2 in.) squares, with the dimensions parallel to the warp or wale direction of the fabrics. It is recommended that the samples be sewn or stapled at the four edges to avoid rolled edges and to assist in obtaining a uniform test result over the entire surface. During each washing cycle, the fabrics were washed in capped bottles containing 200 mL of TTE detergent solution at 30 ± 2 °C for 30 min with intense stirring. Thereafter, the samples were placed in ventilated hood and air-dried to a constant weight. All the samples were conditioned at a relative humidity of 65 ± 2% and a temperature of 20 ± 1 °C for 1 h before being subjected to the conductivity tests.

## Additional Information

**How to cite this article**: Wu, B. *et al*. Electrical Switchability and Dry-Wash Durability of Conductive Textiles. *Sci. Rep*. **5**, 11255; doi: 10.1038/srep11255 (2015).

## Supplementary Material

Supplementary Information

## Figures and Tables

**Figure 1:**
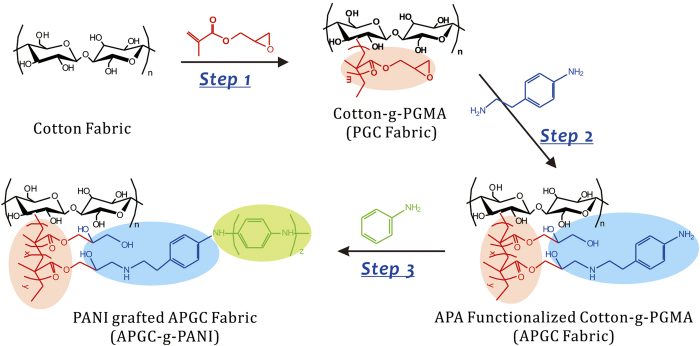
Schematic diagram of conductive textiles preparation by chemical grafting of PANI onto cotton fabrics. ***Step 1***: polymeric graft chains containing epoxy groups were connected to pristine cotton fabric by gamma ray irradiation-induced grafting polymerization of glycidyl methacrylate (GMA). ***Step 2***: 4-aminophenethylamine (APA) is attached to the cotton-g-PGMA (i.e. PGC) fabric via an amino-epoxy ring cleavage reaction. ***Step 3***: PANI is grafted onto APA functionalized PGC (i.e. APGC) fabric by in-situ redox polymerization of aniline.
