# Supplementary material for: Electrical Switchability and Dry-Wash Durability of Conductive Textiles
Source: Sci Rep. 2015 Jun 12;5:11255. doi: 10.1038/srep11255 (PMC4464307; doi:10.1038/srep11255)
Supplement: Supplementary Information [file srep11255-s1.pdf]

**Supplementary Information for**

# Electrical Switchability and Dry-Wash Durability of Conductive Textile

Bangting Wu,[1, 2†] Bowu Zhang,[1†] Jingxia Wu,[1, 2] Ziqiang Wang,[1] Hongjuan Ma,[1] Ming Yu,[1] Linfan Li[1] and Jingye Li[1]*

[1] CAS Center for Excellence on TMSR Energy System, Shanghai Institute of Applied Physics, Chinese Academy of Sciences, Shanghai 201800, China

[2] University of Chinese Academy of Sciences, Beijing 100049, China

Correspondence should be addressed to J. L., E-mail: jingyeli@sinap.ac.cn

†B. W. and B. Z. contributed to this work equally.

## *Experimental Section*

***S1. Chemicals and materials*:** Cotton textiles (100% cotton) were procured from a local supplier in cloth form, and further purified by extraction with acetone for 24 hours in Soxhlet extractor and completely dried in baking oven at 60 ˚C before use. Glycidyl methacrylate (GMA) was purchased from Sigma-Aldrich Co., Ltd. 4-Aminophenethylamine (APA) was purchased from Tokyo Chemical Industry Co., Ltd. Other chemical agents, including aniline, hydrochloric acid (37%), ammonium persulfate ($(NH_4)_2S_2O_8$, APS), tetrachloroethylene, sodium hydroxide, etc. were all of analytical grade and purchased from Sinopharm Chemical Reagent Co., Ltd. All chemical agents were used without further purification unless otherwise stated. Water here used was deionized water.

***S2. Simultaneous radiation induced graft modification of pristine cotton fabric (Step 1)***: Pristine cotton fabric was immerged in 80 ml solution of GMA (5 wt.%) in methanol/water mixture (6:4, v/v) in a glass tube. The tube was deaerated with nitrogen purging for 15 minutes and then sealed with silicon grease. Thereafter, the sealed tube was irradiated in $^{60}$Co source room at a constant dose rate of 1.76 kGy $h^{-1}$ at room temperature (ca. 25 ˚C). After 17 hours, the resulting cotton fabric was extracted for 72 hours by acetone in Soxhlet extractor to remove homopolymer, and then oven-dried at 60 ℃ completely. The obtained fabric was designated as PGC fabric. The degree of grafting (DG) of polyglycidyl methacrylate (PGMA) was determined by gravimetric method and calculated according to equation (1):

$$DG\ of\ PGMA(\%) = \frac{W_1 - W_0}{W_0} \times 100\% \tag{1}$$

Wherein, $W_0$ and $W_1$ represent the weights of pristine cotton and PGC fabric, respectively. The DG of PGMA in this experiment is 25.6 wt.%.

***S3. APA functionalization of PGC fabric (Step 2):*** PGC fabric was immerged in 200 ml of APA solution (0.5 wt.%) with pH at 13 in a capped bottle. Then, the bottle was purged with nitrogen for 15 minutes and sealed. The reaction was carried out in oil bath at 80 ˚C for 24 hours under vigorous magnetic stirring. After that, fabric was first washed with deionized water and subsequently with acetone to remove any excess of reactants, and then oven-dried at 60 ℃ to a constant weight. The obtained fabric from this step was designated as APGC fabric. The conversion rate of epoxy groups on PGC fabric was defined as the molar fraction of attached APA to overall GMA on fabric, and calculated according to equation (2):

$$C(\%) = \left(\frac{W_2 - W_1}{M_2}\right) \Big/ \left(\frac{W_1 - W_0}{M_1}\right) \times 100\% \tag{2}$$

Wherein, $W_0$, $W_1$ *and* $W_2$ represent the weights of pristine cotton, PGC fabric and APGC fabric; $M_1$ and $M_2$ represent the molar mass of GMA and APA, respectively. The conversion rate of epoxy groups in this experiment is 24.7 %.

***S4. In situ redox graft polymerization of aniline on APGC fabric (Step 3):*** APGC fabric was immerged in 400 ml aqueous hydrochloric acid solution. As temperature was equilibrated in ice-water bath, the polymerization was initiated by adding 100 ml aniline solution drop-wise, with a speed of 25 ml $h^{-1}$. Equal amount of APS solution was simultaneously dropped into the reactor as the same way in

same molarity. The reaction was carried out under constant nitrogen flux and temperature for 24 hours with intense mechanical stirring. Then the fabric was scrubbed in water several times and extracted for 72 hours by chloroform in Soxhlet extractor. After that, the obtained fabric was dried in 60 ℃ vacuum oven overnight. The resultant fabric here was designated as APGC-g-PANI fabric. The DG of PANI was also determined by gravimetric method and calculated according to equation (3):

$$DG\ of\ PANI\ (\%) = \frac{W_3 - W_2}{W_0} \times 100\% \qquad (3)$$

Wherein, $W_0$, $W_2$ *and* $W_3$ represent the weights of pristine cotton, APGC fabric and APGC-g-PANI, respectively.

***S5. General Characterizations*:** Bruker Optics TENSOR 27 FT-IR Spectrometer with horizontal multi-rebound attenuated total reflection (ATR) was used to characterize the obtained fabrics in the wavenumber range of 4000-600 $cm^{-1}$. Spectra were recorded at 32 scans average and 4 $cm^{-1}$ resolution. X-ray Photoelectron Spectroscopy (XPS) analysis was carried out on a SHIMADZU Kratos AXIS Ultra DLD XPS instrument equipped with a monochromated Al K α (1486.6 eV) X-ray source. Survey scans of the samples were carried out in the 0-1100 eV range, and detailed scans were performed for the C1s and N1s regions. Hitachi S-4800 field emission scanning electron microscope (FESEM) was used for the surface morphological characterization of the fabric samples, which were sputter-coated with gold and examined at an acceleration voltage of 10 kV. The static water contact angles (CA) were determined on a KSV ATTENSION Theta Optical Tensionmeter. A 5 μL water drop from needle tip was stroked onto the sample surface. Shape of the droplet was recorded by a digital camera and static contact angle was calculated according to images taken by evaluation software provided from the instrument manufacturer.

**Table S1** The elemental analysis of pristine cotton fabric, PGC fabric, APGC fabric and APGC-g-PANI fabric by XPS.

| | **C**(wt.%) | **O**(wt.%) | **N**(wt.%) |
|---|---|---|---|
| **Cotton fabric[1]** | 54.75 | 45.25 | —— |
| **PGC fabric[2]** | 59.86 | 40.14 | —— |
| **APGC fabric[3]** | 68.31 | 25.51 | 6.19 |
| **APGC-g-PANI fabric[4]** | 63.85 | 25.29 | 8.23 |

[1] Cotton fabric was extracted by actone in Soxhlet extractor before testing;

[2] PGC fabric with 25.6 wt.% DG of PGMA;

[3] APGC fabric with 24.7 % conversion of epoxy group;

[4] APGC-g-PANI fabric with 7.3 wt.% DG of PANI.

# Figures

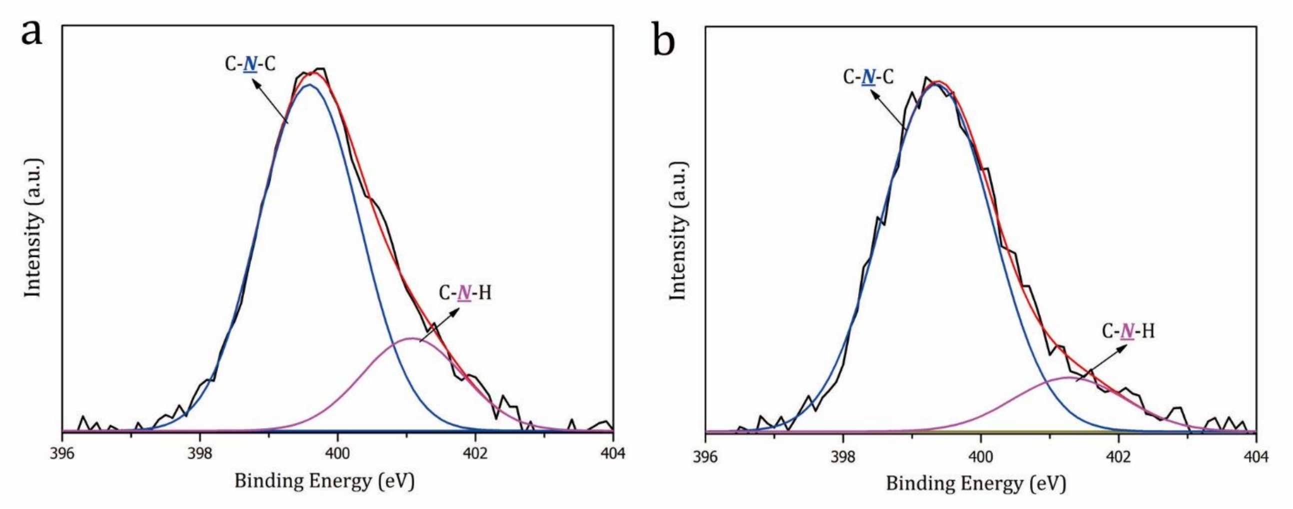


**Figure S1** N1s XPS spectra of (a) APGC fabric and (b) APGC-g-PANI fabric.

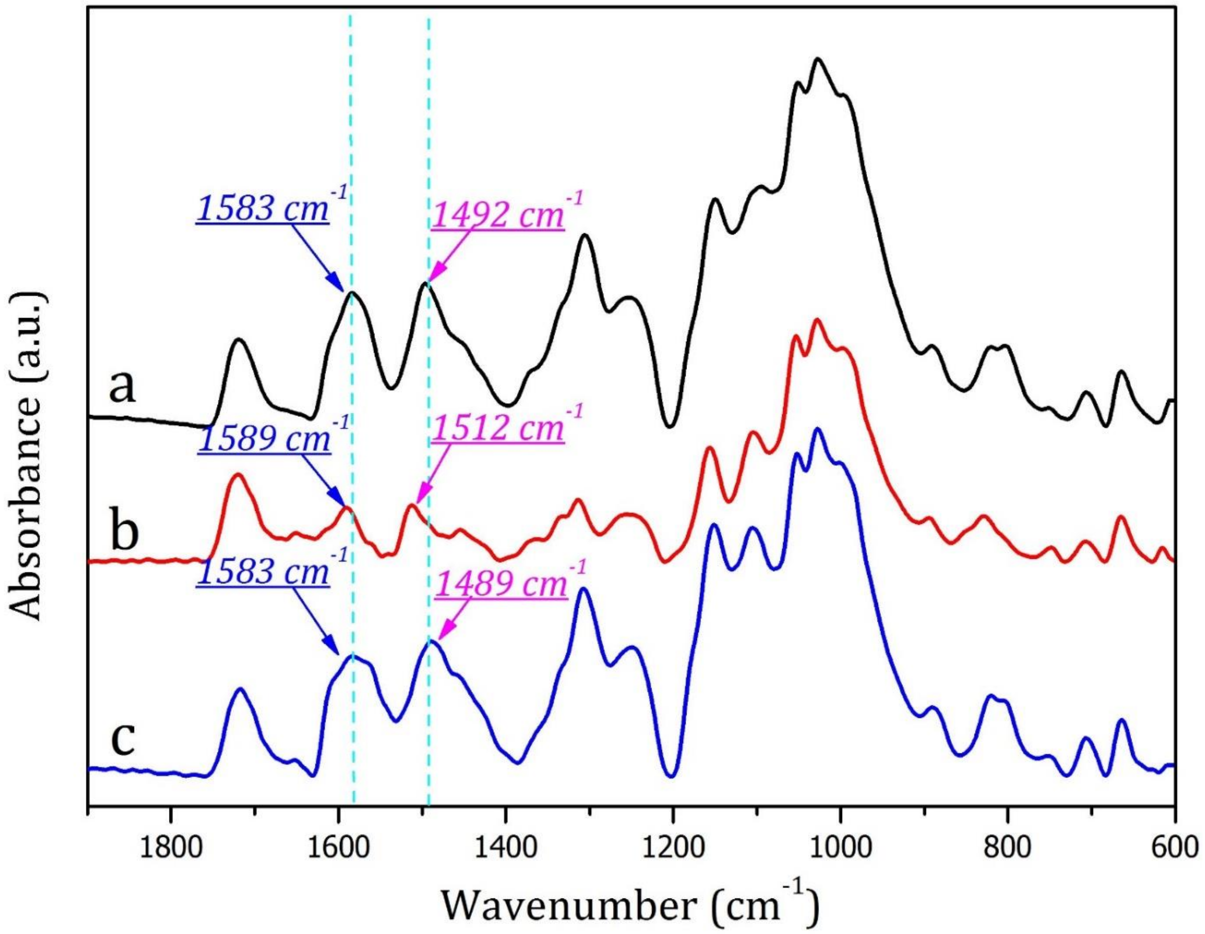


**Figure S2** The FTIR-ATR spectra of (a) as-prepared APGC-g-PANI fabric, after immersed in (b) alkaline (pH=14) and (c) acidic (pH=0) bath solution.

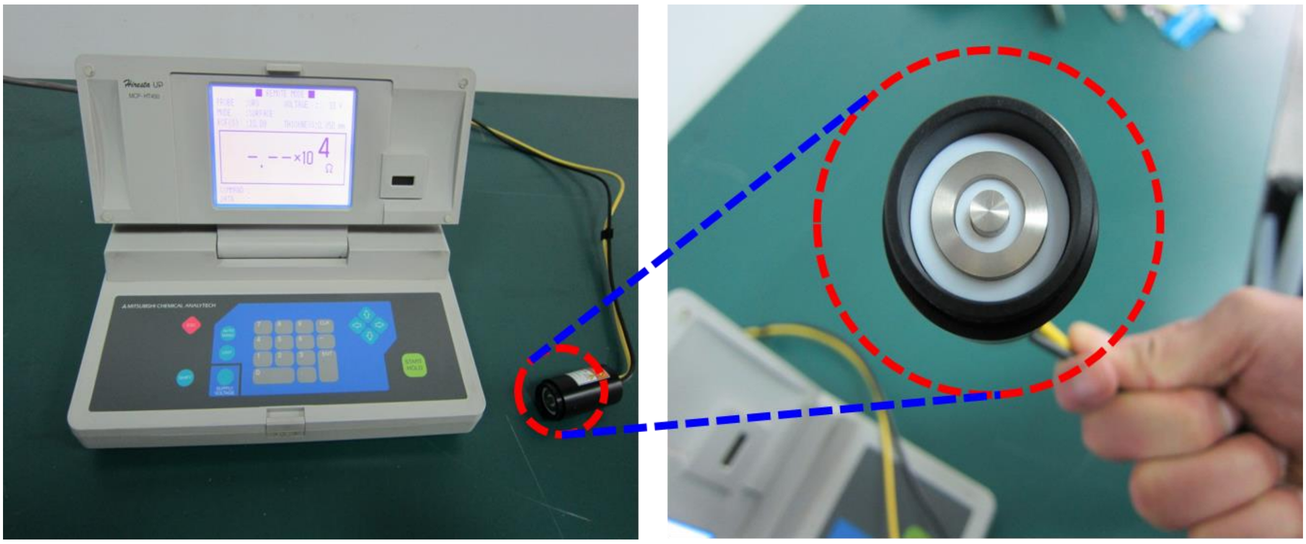

**Figure S3** The ultrahigh-resistivity meter (Hiresta-UP MCP-HT450) and its concentric ring probes.
